# Supplementary material for: Measures of Maximal Tactile Pressures during a Sustained Grasp Task Using a TactArray Device Have Satisfactory Reliability and Concurrent Validity in People with Stroke
Source: Sensors (Basel). 2023 Mar 20;23(6):3291. doi: 10.3390/s23063291 (PMC10059963; doi:10.3390/s23063291)
Supplement: Supplementary file 1 [file sensors-23-03291-s001.zip › sensors-2235764-supplementary.pdf]

Supplementary Tables

Table S1. Measures of reliability in the affected hand of participants with stroke without vision during complete grasp duration (8 s)

		Session 1	Session 2	Session 3	Mean 3 sessions		Change in mean (%)	90% CI for change in mean	CV (%)	90% CI for change in CV	Smallest effect (%)*	90% CI for change in smallest effect	ICC	90% CI for change in ICC
Pressure/kPa														
Pres(8s)max	Mean	32.02	33.69	31.81	32.50	Session 2-1	11.15	-2.52, 26.73	18.50	13.37, 31.06	8.36	2.98, 11.59	0.88	0.68, 0.96
	SD	14.64	10.82	12.01	12.59	Session 3-2	-7.08	-17.18, 4.24	16.05	11.63, 26.75	7.06	2.46, 9.78	0.87	0.66, 0.95
Pres(8s)avg2	Mean	30.05	31.10	29.85	30.34	Session 2-1	8.10	-5.52, 23.68	19.03	13.74, 31.99	9.63	3.74, 13.29	0.90	0.73, 0.97
	SD	14.40	11.51	11.27	12.47	Session 3-2	-3.48	-14.59, 9.08	17.15	12.41, 28.68	8.04	2.97, 11.12	0.89	0.69, 0.96
Pres(8s)avg3	Mean	29.56	30.69	29.69	29.98	Session 2-1	9.38	-4.02, 24.65	18.42	13.31, 30.91	8.99	3.41, 12.41	0.89	0.71, 0.96
	SD	13.97	10.46	10.88	11.87	Session 3-2	-3.66	-13.36, 7.13	14.72	10.69, 24.46	7.55	2.97, 10.37	0.90	0.73, 0.97
Force/N														
Force(8s)max	Mean	49.46	47.58	42.58	46.54	Session 2-1	0.86	-13.65, 17.81	22.26	16.01, 37.74	11.91	4.76, 16.44	0.91	0.76, 0.97
	SD	25.53	21.03	14.65	20.88	Session 3-2	-7.39	-16.80, 3.08	14.87	10.79, 24.71	9.91	4.34, 13.51	0.94	0.83, 0.98
Force(8s)avg 2	Mean	40.01	46.07	39.99	42.02	Session 2-1	18.68	3.72, 35.79	19.04	13.75, 32.01	11.61	4.91, 15.92	0.93	0.80, 0.98
	SD	17.86	20.06	13.83	17.44	Session 3-2	-10.95	-18.20, -3.05	11.62	8.46, 19.14	10.11	4.69, 13.69	0.96	0.89, 0.99
Force(8s)avg 3	Mean	42.21	45.08	38.85	42.04	Session 2-1	11.31	-4.45, 29.67	21.84	15.72, 36.98	11.69	4.68, 16.13	0.91	0.76, 0.97
	SD	20.22	19.51	13.02	17.88	Session 3-2	-10.91	-19.41, -1.51	13.86	10.07, 22.96	9.81	4.36, 13.34	0.94	0.84, 0.98

*:smallest effect from pure SD; CI: Confidence interval: CV: Coefficient of variation; SD: standard deviation

Table S2. Measures of reliability in the affected hand of participants with stroke with vision during the plateau phase (5 s)

		Session 1	Session 2	Session 3	Mean 3 sessions		Change in mean (%)	90% CI for change in mean	CV (%)	90% CI for change in CV	Smallest effect (%)*	90% CI for change in smallest effect	ICC	90% CI for change in ICC
**Pressure/kPa**														
Pres(5s)max	Mean	39.33	41.26	41.00	40.53	Session 2-1	4.35	-7.28, 17.43	16.52	11.96, 27.57	10.40	4.46, 14.22	0.93	0.81, 0.98
	SD	15.20	14.66	13.92	14.60	Session 3-2	3.94	-14.38, 26.17	28.51	20.37, 49.13	7.87	-1.79, 11.48	0.74	0.38, 0.90
						Session 3-2#	-5.68	-13.89, 3.31	11.74	8.44, 20.04	9.15	4.17, 12.39	0.95	0.86, 0.98
Pres(5s)avg2	Mean	35.17	39.64	37.13	37.31	Session 2-1	11.92	-0.65, 26.07	16.66	12.07, 27.83	10.67	4.60, 14.59	0.93	0.82, 0.98
	SD	14.29	14.99	10.57	13.43	Session 3-2	0.16	-17.61, 21.75	28.74	20.53, 49.54	7.61	-2.09, 11.17	0.72	0.35, 0.90
						Session 3-2#	-9.48	-16.02, -2.42	9.58	6.90, 16.25	9.13	4.31, 12.32	0.97	0.90, 0.99
Pres(5s)avg3	Mean	36.07	38.27	37.87	37.40	Session 2-1	6.19	-5.04, 18.76	15.57	11.29, 25.92	10.75	4.74, 14.66	0.94	0.84, 0.98
	SD	14.65	13.95	11.81	13.52	Session 3-2	4.44	-13.93, 26.73	28.44	20.32, 48.99	7.76	-1.88, 11.33	0.74	0.38, 0.90
						Session 3-2#	-5.45	-12.57, 2.25	10.02	7.21, 17.01	9.17	4.30, 12.38	0.97	0.89, 0.99
**Force/N**														
Force(5s)max	Mean	56.91	58.50	54.54	56.65	Session 2-1	7.09	-4.28, 19.80	15.63	11.33, 26.02	12.78	5.83, 17.40	0.96	0.88, 0.99
	SD	28.76	23.66	15.01	23.18	Session 3-2	-0.31	-13.43, 14.79	20.03	14.44, 33.75	9.02	3.21, 12.52	0.88	0.68, 0.96
Force(5s)avg2	Mean	49.06	53.54	48.96	50.52	Session 2-1	11.05	0.85, 22.27	13.27	9.64, 21.95	14.50	6.85, 19.68	0.98	0.93, 0.99
	SD	24.13	24.33	15.88	21.81	Session 3-2	-0.32	-16.83, 19.47	26.40	18.91, 45.25	10.60	3.33, 14.88	0.86	0.62, 0.95
Force(5s)avg3	Mean	50.38	53.91	49.74	51.34	Session 2-1	9.78	0.19, 20.29	12.56	9.14, 20.74	13.70	6.48, 18.57	0.97	0.93, 0.99
	SD	25.20	23.47	15.11	21.71	Session 3-2	-0.11	-15.76, 18.44	24.67	17.70, 42.08	9.74	2.97, 13.67	0.85	0.61, 0.95

#:outlier removed; *:smallest effect from pure SD; CI: Confidence interval: CV: Coefficient of variation; SD: standard deviation

Table S3. Measures of reliability in the affected hand of participants with stroke without vision during the plateau phase (5 s)

		Session 1	Session 2	Session 3	Mean 3 sessions		Change in mean (%)	90% CI for change in mean	CV (%)	90% CI for change in CV	Smallest effect (%)*	90% CI for change in smallest effect	ICC	90% CI for change in ICC
Pressure/kPa														
Pres(5s)max	Mean	39.90	41.80	39.48	40.39	Session 2-1	11.10	-2.86, 27.06	18.97	13.70, 31.89	8.89	3.27, 12.30	0.89	0.69, 0.96
	SD	19.12	14.42	14.48	16.15	Session 3-2	-6.30	-16.61, 5.29	16.29	11.80, 27.17	7.27	2.57, 10.07	0.87	0.67, 0.96
Pres(5s)avg2	Mean	36.65	38.32	36.90	37.29	Session 2-1	9.65	-2.90, 23.83	17.04	12.33, 28.48	10.20	4.30, 13.97	0.93	0.79, 0.97
	SD	18.72	14.93	12.70	15.65	Session 3-2	-1.43	-12.84, 11.46	17.24	12.48, 28.85	7.91	2.86, 10.94	0.88	0.68, 0.96
Pres(5s)avg3	Mean	36.45	37.74	36.91	37.04	Session 2-1	9.35	-4.42, 25.11	19.03	13.74, 31.99	9.35	3.56, 12.91	0.89	0.72, 0.96
	SD	17.69	13.39	13.29	14.93	Session 3-2	-2.07	-11.61, 8.50	14.18	10.30, 23.52	7.76	3.16, 10.63	0.91	0.76, 0.97
Force/N														
Force(5s)max	Mean	61.56	59.60	53.46	58.21	Session 2-1	1.92	-13.40, 19.94	23.45	16.85, 39.88	12.36	4.90, 17.08	0.91	0.75, 0.97
	SD	33.38	27.52	19.11	27.30	Session 3-2	-6.97	-16.86, 4.09	15.65	11.35, 26.07	10.30	4.48, 14.05	0.94	0.82, 0.98
Force(5s)avg2	Mean	50.78	57.35	50.23	52.78	Session 2-1	16.06	0.90, 33.49	19.85	14.32, 33.44	12.26	5.21, 16.82	0.93	0.81, 0.98
	SD	23.67	26.69	18.42	23.18	Session 3-2	-9.48	-16.94, -1.36	11.77	8.57, 19.39	10.70	4.99, 14.49	0.96	0.90, 0.99
Force(5s)avg3	Mean	52.58	56.00	48.81	52.46	Session 2-1	11.14	-5.14, 30.22	22.75	16.36, 38.62	12.07	4.80, 16.67	0.91	0.75, 0.97
	SD	26.02	25.36	16.92	23.14	Session 3-2	-9.60	-18.26, -0.03	13.91	10.10, 23.06	10.15	4.55, 13.80	0.95	0.85, 0.98

*:smallest effect from pure SD; CI: Confidence interval: CV: Coefficient of variation; SD: standard deviation

Table S4. Measures of reliability in the less affected hand of participants with stroke without vision during complete grasp duration (8 s)

		Session 1	Session 2	Session 3	Mean 3 sessions		Change in mean (%)	90% CI for change in mean	CV (%)	90% CI for change in CV	Smallest effect (%)*	90% CI for change in smallest effect	ICC	90% CI for change in ICC
Pressure/kPa														
Pres(8s)max	Mean	36.44	37.25	35.10	36.26	Session 2-1	7.36	-3.03, 18.87	14.08	10.23, 23.36	10.37	4.66, 14.11	0.95	0.85, 0.98
	SD	17.37	13.49	8.99	13.71	Session 3-2	-1.78	-12.48, 10.24	16.11	11.67, 26.86	7.20	2.55, 9.98	0.87	0.67, 0.96
Pres(8s)avg2	Mean	34.49	36.07	32.09	34.22	Session 2-1	11.67	-0.57, 25.41	16.21	11.74, 27.03	10.82	4.72, 14.77	0.94	0.83, 0.98
	SD	17.68	13.16	9.79	13.93	Session 3-2	-8.70	-15.79, -1.02	11.02	8.03, 18.12	8.25	3.74, 11.18	0.95	0.86, 0.98
Pres(8s)avg3	Mean	34.16	34.37	32.47	33.67	Session 2-1	6.94	-5.07, 20.48	16.67	12.07, 27.84	10.46	4.48, 14.30	0.93	0.81, 0.98
	SD	17.15	12.12	8.93	13.17	Session 3-2	-2.38	-10.68, 6.69	12.19	8.87, 20.11	7.72	3.34, 10.50	0.93	0.81, 0.98
Force/N														
Force(8s)max	Mean	46.39	50.16	44.49	47.01	Session 2-1	4.45	-7.46, 17.89	16.96	12.28, 28.35	10.25	4.34, 14.04	0.93	0.80, 0.97
	SD	17.93	25.30	13.57	19.54	Session 3-2	-5.02	-17.35, 9.17	19.73	14.23, 33.22	9.02	3.25, 12.50	0.88	0.68, 0.96
Force(8s)avg2	Mean	42.79	47.82	40.85	43.82	Session 2-1	11.52	-2.13, 27.07	18.40	13.30, 30.87	11.23	4.76, 15.39	0.93	0.80, 0.98
	SD	20.28	24.68	12.71	19.85	Session 3-2	-8.24	-20.54, 5.97	20.47	14.75, 34.53	9.20	3.27, 12.78	0.88	0.68, 0.96
Force(8s)avg3	Mean	42.78	45.25	40.86	42.96	Session 2-1	5.04	-5.96, 17.34	15.40	11.16, 25.63	10.56	4.65, 14.40	0.94	0.84, 0.98
	SD	18.64	21.47	12.33	17.89	Session 3-2	-4.09	-15.62, 9.01	18.02	13.03, 30.21	8.86	3.38, 12.22	0.89	0.72, 0.96

*:smallest effect from pure SD; CI: Confidence interval: CV: Coefficient of variation; SD: standard deviation

Table S5. Measures of reliability in the less affected hand of participants with stroke with vision during the plateau phase (5 s)

		Session 1	Session 2	Session 3	Mean 3 sessions		Change in mean (%)	90% CI for change in mean	CV (%)	90% CI for change in CV	Smallest effect (%)*	90% CI for change in smallest effect	ICC	90% CI for change in ICC
Pressure/kPa														
Pres(5s)max	Mean	50.06	44.39	45.23	46.56	Session 2-1	-8.96	-26.80, 13.24	32.62	23.20, 56.79	7.34	-3.13, 11.08	0.66	0.24, 0.87
	SD	23.38	14.81	13.22	17.71	Session 3-2	4.34	-8.05, 18.40	17.77	12.85, 29.77	7.38	2.40, 10.28	0.86	0.63, 0.95
Pres(5s)avg2	Mean	48.56	42.92	42.64	44.70	Session 2-1	-8.87	-27.71, 14.89	34.95	24.80, 61.20	7.17	-3.63, 11.03	0.62	0.18, 0.85
	SD	23.12	14.65	12.58	17.39	Session 3-2	1.96	-9.51, 14.89	16.70	12.09, 27.89	7.33	2.54, 10.17	0.87	0.66, 0.95
Pres(5s)avg3	Mean	48.06	41.69	42.90	44.22	Session 2-1	-10.51	-27.98, 11.19	32.44	23.08, 56.45	7.27	-3.13, 10.98	0.66	0.24, 0.87
	SD	22.90	13.76	12.89	17.12	Session 3-2	5.04	-6.30, 17.75	15.92	11.54, 26.54	7.38	2.70, 10.20	0.88	0.69, 0.96
Force/N														
Force(5s)max	Mean	62.39	55.20	60.84	59.48	Session 2-1	-11.95	-25.58, 4.18	24.32	17.45, 41.45	7.77	0.97, 11.11	0.79	0.48, 0.92
	SD	22.45	19.99	17.67	20.13	Session 3-2	13.40	1.28, 26.97	15.75	11.41, 26.24	7.73	2.95, 10.64	0.89	0.71, 0.96
Force(5s)avg2	Mean	57.13	50.33	56.72	54.73	Session 2-1	-13.17	-26.94, 3.20	25.04	17.96, 42.76	7.75	0.32, 11.12	0.78	0.46, 0.92
	SD	20.95	19.78	18.16	19.66	Session 3-2	15.79	3.89-29.06	15.07	10.93, 25.05	8.24	3.35, 11.29	0.91	0.76, 0.97
Force(5s)avg3	Mean	57.06	50.59	56.51	54.72	Session 2-1	-11.18	-25.20, 5.46	24.88	17.85, 42.47	7.85	0.82, 11.25	0.78	0.47, 0.92
	SD	21.30	18.49	17.83	19.27	Session 3-2	14.14	3.26, 26.16	13.84	10.05, 22.93	7.96	3.32, 10.88	0.92	0.78, 0.97

*:smallest effect from pure SD; CI: Confidence interval: CV: Coefficient of variation; SD: standard deviation

Table S6. Measures of reliability in the less affected hand of participants with stroke without vision during the plateau phase (5 s)

		Session 1	Session 2	Session 3	Mean 3 sessions		Change in mean (%)	90% CI for change in mean	CV (%)	90% CI for change in CV	Smallest effect (%)*	90% CI for change in smallest effect	ICC	90% CI for change in ICC
Pressure/kPa														
Pres(5s)max	Mean	44.72	45.41	43.46	44.53	Session 2-1	7.16	-3.69, 19.24	14.82	10.75, 24.63	10.67	4.76, 14.53	0.95	0.85, 0.98
	SD	21.18	16.38	11.23	16.76	Session 3-2	-0.15	-10.99, 12.01	16.03	11.62, 26.73	7.28	2.62, 10.08	0.88	0.68, 0.96
Pres(5s)avg2	Mean	41.86	42.78	39.81	41.48	Session 2-1	7.63	-3.27, 19.75	14.81	10.75, 24.61	10.89	4.88, 14.82	0.95	0.85, 0.98
	SD	20.98	15.94	12.07	16.73	Session 3-2	-3.80	-12.59, 5.86	13.19	9.59, 21.82	8.41	3.65, 11.46	0.93	0.81, 0.98
Pres(5s)avg3	Mean	41.56	41.52	40.15	41.08	Session 2-1	6.10	-5.42, 19.01	16.03	11.61, 26.72	10.52	4.57, 14.36	0.94	0.82, 0.98
	SD	20.57	14.64	11.07	15.92	Session 3-2	0.00	-8.43, 9.20	12.06	8.78, 19.89	7.72	3.36, 10.50	0.93	0.81, 0.98
Force/N														
Force(5s)max	Mean	56.69	60.92	54.89	57.50	Session 2-1	3.95	-8.40, 17.97	17.78	12.86, 29.79	10.34	4.30, 14.18	0.92	0.78, 0.97
	SD	22.45	30.86	17.31	24.19	Session 3-2	-3.50	-15.99, 10.85	19.64	14.17, 33.07	9.09	3.31, 12.60	0.88	0.69, 0.96
Force(5s)avg2	Mean	52.36	56.70	51.11	53.39	Session 2-1	6.98	-7.25, 23.39	20.28	14.62, 34.20	11.45	4.70, 15.75	0.92	0.77, 0.97
	SD	24.70	30.51	15.35	24.33	Session 3-2	-1.47	-15.47, 14.84	21.93	15.78, 37.15	9.19	3.03, 12.83	0.86	0.64, 0.95
Force(5s)avg3	Mean	52.19	54.87	50.60	52.56	Session 2-1	4.23	-7.17, 17.04	16.18	11.72, 26.98	10.55	4.58, 14.41	0.94	0.82, 0.98
	SD	22.79	26.42	15.65	22.08	Session 3-2	-1.98	-13.71, 11.35	17.93	12.97, 30.06	8.90	3.42, 12.28	0.90	0.72, 0.96

*:smallest effect from pure SD; CI: Confidence interval: CV: Coefficient of variation; SD: standard deviation
